# Systemic immune-inflammation index is associated with diabetic peripheral neuropathy: evidence from NHANES, an external clinical cohort, and exploratory human nerve gene-expression analysis

**DOI:** 10.3389/fendo.2026.1948081

**Published:** 2026-09-07

**Authors:** Chenchen Kong, Zhenxuan Gao, Quanyu Jin, Ze Zhang, Jiaxin Liu, Lei Kou, Aerman Abudurezhake, Shuguang Cai, Ge Shi, Li Zhang

**Affiliations:** 1 China-Japan Friendship Hospital, Chinese Academy of Medical Sciences & Peking Union Medical College, Beijing, China; 2 Peking University China-Japan Friendship School of Clinical Medicine, Beijing, China; 3 China-Japan Friendship Institute of Clinical Medicine, Beijing, China; 4 Department of Neurosurgery, The Second Affiliated Hospital of Soochow University, Suzhou, China; 5 Department of Neurosurgery, China-Japan Friendship Hospital, Beijing, China

**Keywords:** BDNF, c-Jun, diabetic peripheral neuropathy, gene expression, NHANES, platelets, systemic immune-inflammation index

## Abstract

**Background:**

The association between the systemic immune-inflammation index (SII) and diabetic peripheral neuropathy (DPN) remains inconsistent across studies. We investigated the association between SII and prevalent DPN across independent populations and explored complementary biological context using human sural nerve gene-expression data.

**Methods:**

We analyzed a discovery cohort of 555 adults with diabetes from the National Health and Nutrition Examination Survey (NHANES) 1999–2000 and performed an exploratory targeted gene-expression analysis of human sural nerve samples from GSE95849. Findings were further evaluated in an independent hospital-based cohort of 400 patients with type 2 diabetes mellitus (T2DM), including 290 with DPN and 110 without DPN. Multivariable logistic regression, component analyses, sensitivity analyses, and restricted cubic spline models were used to evaluate the association between SII and prevalent DPN.

**Results:**

In NHANES, higher SII was inversely associated with prevalent DPN. Compared with participants in the lowest SII quartile, those in the highest quartile had lower odds of DPN (odds ratio [OR] = 0.239, 95% confidence interval [CI]: 0.102–0.560; *P* < 0.001). Exploratory targeted analysis of sural nerve tissue showed significantly lower *BDNF* expression in DPN samples than in samples from patients with diabetes without DPN (BH-adjusted *P* = 0.0004). *JUN* expression was also lower in DPN at the nominal level, but the difference did not remain statistically significant after multiple-comparison correction (BH-adjusted *P* = 0.0686), whereas *PDGFRA* expression did not differ between groups. In the external cohort, patients with DPN had lower platelet counts than those without DPN (81.6 vs. 135.9 × 10^9^/L; *P* < 0.001). SII remained inversely associated with prevalent DPN after multivariable adjustment (OR = 0.396 per 100-unit increase, 95% CI: 0.304–0.516; *P* < 0.001). Restricted cubic spline analysis indicated a significant nonlinear association (P for nonlinearity < 0.001). Among the hematological components examined, platelet count showed the strongest and most consistent association with DPN, whereas the neutrophil-to-lymphocyte ratio (NLR) was no longer significant after mutual adjustment.

**Conclusions:**

Lower SII was consistently associated with prevalent DPN across two independent populations, with a significant nonlinear relationship observed in the external cohort. Platelet count showed the strongest and most consistent association among the hematological components examined. Exploratory nerve-tissue gene-expression findings provide complementary biological context but do not establish a mechanistic or causal link between platelet-related hematological indices and DPN.

## 1 Background

Diabetic peripheral neuropathy (DPN) is one of the most common and debilitating microvascular complications of diabetes, affecting up to 50% of patients during their lifetime (1–3). Characterized by progressive sensory loss, pain, and an increased risk of foot ulceration, DPN imposes a profound socioeconomic burden. The pathogenesis of DPN is complex and involves chronic hyperglycemia-related metabolic, oxidative, and microvascular disturbances (4, 5). However, progressive neuropathy may occur despite glycemic management, suggesting that additional vascular, neurotrophic, and immune-inflammatory processes may also be relevant (6, 7).

In recent years, the systemic immune-inflammation index (SII)—a composite hematological index calculated from platelet, neutrophil, and lymphocyte counts—has been investigated in a range of vascular and metabolic conditions (8, 9). Higher SII is commonly interpreted as reflecting a greater systemic inflammatory burden (10), and several studies have reported positive associations between higher SII and diabetic complications, including DPN (6, 11, 12). Diabetes is a progressive and heterogeneous disease characterized by changes in metabolic, cellular, and immune function over time (13, 14). Such disease-stage-related changes raise the possibility that systemic inflammatory and hematological profiles may also vary during the course of diabetes and its complications. However, whether SII follows a stage-dependent trajectory in DPN remains uncertain.

Platelets have biological functions beyond hemostasis and inflammation, including the storage and release of growth factors such as brain-derived neurotrophic factor (BDNF) (15, 16). These established platelet functions provide biological context for examining the platelet component of SII in relation to peripheral nerve disease. Nevertheless, platelet count does not directly measure platelet function, circulating BDNF availability, or neurotrophic capacity, and a causal platelet-mediated pathway cannot be inferred from hematological associations alone.

Accordingly, we designed a multidimensional study to characterize the association between SII and prevalent DPN across complementary datasets. First, we evaluated the epidemiological association using the NHANES 1999–2000 cohort. Second, we performed an exploratory targeted gene-expression analysis of human sural nerve samples from the Gene Expression Omnibus (GEO) to provide complementary biological context. Finally, we examined the reproducibility of the SII–DPN association in an independent hospital-based cohort of patients with T2DM. This design was intended to assess the consistency of the observed association and generate biological hypotheses rather than establish causality, diagnostic performance, or a specific molecular mechanism.

## 2 Materials and methods

### 2.1 Study design and data sources

**Figure 1:**
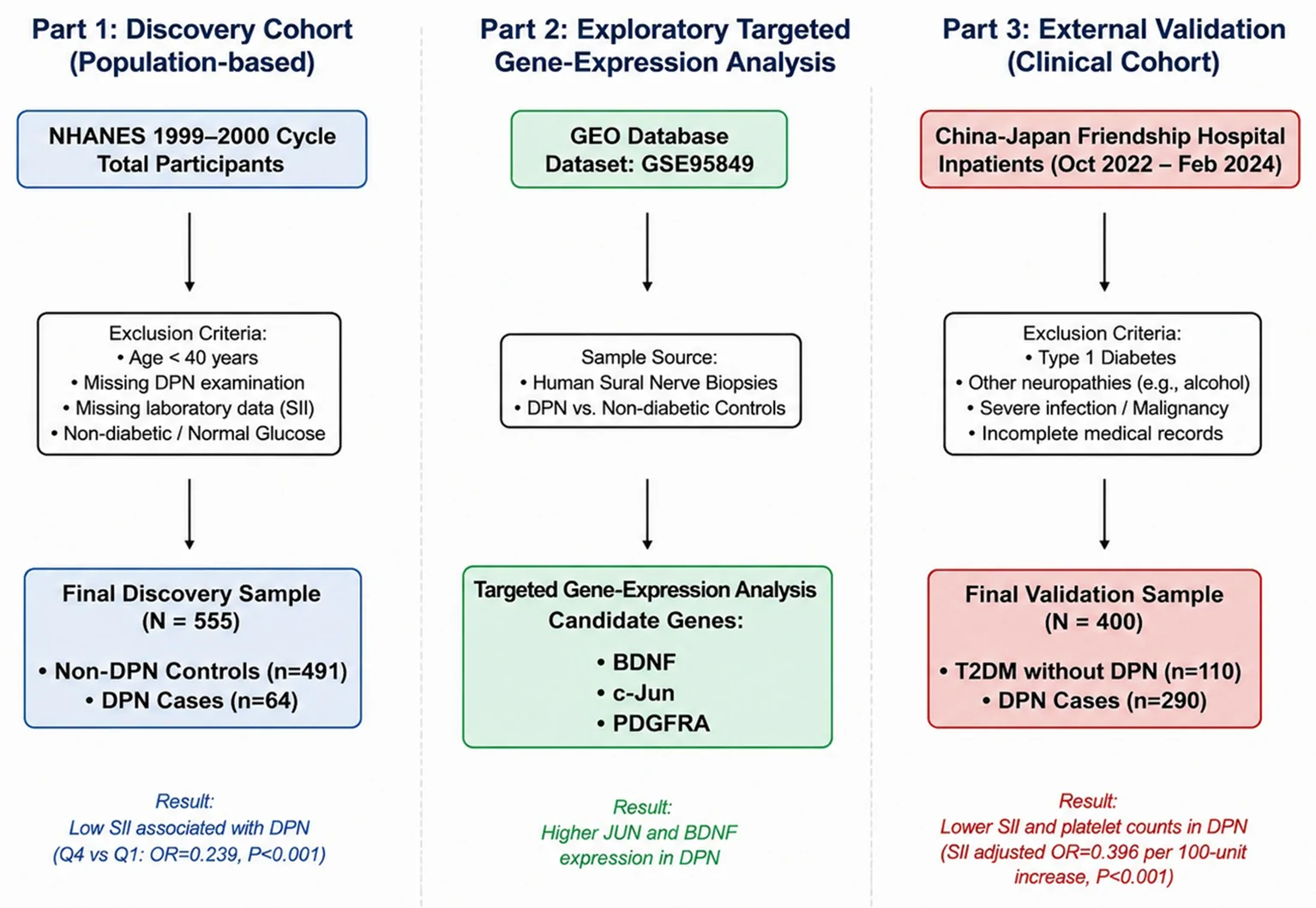
Study flowchart of the multi-population analysis. The study design comprised three complementary components: (1) Discovery analysis: a cross-sectional analysis of 555 adults with diabetes from the NHANES 1999–2000 cycle (64 with DPN and 491 without DPN); (2) Exploratory gene-expression analysis: targeted analysis of *JUN*, *BDNF*, and *PDGFRA* in human sural nerve samples from the GEO dataset GSE95849 (6 DPN samples and 6 samples from patients with diabetes without DPN); and (3) External clinical cohort: retrospective analysis of 400 patients with T2DM from China-Japan Friendship Hospital (290 with DPN and 110 without DPN). DPN, diabetic peripheral neuropathy; GEO, Gene Expression Omnibus; NHANES, National Health and Nutrition Examination Survey; SII, systemic immune-inflammation index; T2DM, type 2 diabetes mellitus.

This study employed a multidimensional approach combining a large-scale cross-sectional analysis, an exploratory targeted gene-expression analysis, and an independent external clinical cohort analysis. The overall design and flowchart of this multi-population study are illustrated in **Figure 1**. For the discovery cohort, data were extracted from the National Health and Nutrition Examination Survey (NHANES) 1999–2000 cycle. To provide exploratory biological context, we used human sural nerve gene-expression data from the GSE95849 dataset in the Gene Expression Omnibus (GEO). The external clinical cohort was retrospectively assembled from 400 inpatients with type 2 diabetes mellitus (T2DM) admitted to the Department of Neurosurgery at China-Japan Friendship Hospital between October 2022 and February 2024. The clinical study protocol was approved by the Ethics Committee of China-Japan Friendship Hospital and adhered to the principles of the Declaration of Helsinki.

### 2.2 Study population

#### 2.2.1 NHANES discovery cohort

Participants from the NHANES 1999–2000 cycle were eligible for inclusion if they were aged 40 years or older, met the study definition of diabetes, had an evaluable lower-extremity monofilament examination, and had available platelet, neutrophil, and lymphocyte measurements. Diabetes was operationally defined as meeting at least one of the following criteria: a self-reported physician diagnosis of diabetes, current use of insulin or oral glucose-lowering medication, fasting plasma glucose ≥126 mg/dL, or glycated hemoglobin (HbA1c) ≥6.5%.

Participants with missing information on SII or monofilament-defined DPN status were excluded. Model-specific complete-case analyses were used for the regression analyses. The final discovery cohort comprised 555 participants, including 64 participants with DPN and 491 participants without DPN.

#### 2.2.2 External clinical cohort

The external clinical cohort was retrospectively assembled by consecutively screening patients with T2DM admitted to China-Japan Friendship Hospital between October 2022 and February 2024. Eligible patients were aged ≥18 years, had a confirmed diagnosis of T2DM, and had the clinical and laboratory data required for the present analyses. Complete blood-cell counts used to calculate SII were obtained from the first routine blood sample collected at hospital admission.

Patients with type 1 diabetes mellitus, severe acute infection, hematological disease, malignancy, recent use of medications known to substantially affect blood-cell counts, or peripheral neuropathy attributable to causes other than diabetes were excluded. Alternative causes considered included excessive alcohol exposure, vitamin B12 deficiency, thyroid dysfunction, radiculopathy, spinal stenosis, toxic neuropathy, autoimmune disease, and other neurological disorders where documented.

The final analytic cohort comprised 400 patients with T2DM, including 290 patients with DPN and 110 patients without DPN. No individuals without T2DM were included as controls. Because this was a retrospective study based on all eligible patients identified during the predefined study period, no *a priori* sample-size calculation was performed.

### 2.3 Assessment of diabetic peripheral neuropathy

In the NHANES cohort, DPN was operationally defined as the presence of at least one insensate site on either foot among participants with diabetes aged 40 years or older. Peripheral sensation was assessed by trained health technicians using a standard 5.07 Semmes–Weinstein nylon monofilament delivering approximately 10 g of force. Three plantar sites on each foot—the hallux, first metatarsal head, and fifth metatarsal head—were examined. A site was classified as insensate when the participant provided two incorrect responses, two “unable to determine” responses, or one incorrect and one “unable to determine” response across repeated trials. Participants without an evaluable monofilament examination were excluded. No questionnaire-based neuropathy variable was included in the operational definition of DPN.

In the external clinical cohort, DPN status was ascertained from pre-existing clinical diagnoses documented in the electronic medical records by the treating clinicians. These diagnoses were based on neuropathic symptoms and/or neurological signs consistent with distal symmetric polyneuropathy, after consideration and exclusion of more plausible alternative causes of peripheral neuropathy. Relevant neuropathic symptoms included numbness, tingling, burning pain, and electric-shock-like pain. Neurological findings documented in the medical records could include abnormalities in distal sensation, monofilament sensation, vibration perception, pinprick sensation, or ankle reflexes.

Patients with a documented clinical diagnosis of DPN were classified as the DPN group. Patients with T2DM without a documented diagnosis or clinical evidence of DPN at the time of evaluation were classified as the non-DPN group. Nerve conduction studies were not systematically performed in all participants, and the Toronto Clinical Neuropathy Score and Michigan Neuropathy Screening Instrument were not uniformly applied. SII was calculated retrospectively and was not used to determine DPN status.

### 2.4 Clinical and laboratory measurements

Demographic characteristics, medical history, diabetes duration, smoking status, alcohol consumption, comorbidities, anthropometric measurements, and laboratory results were collected.

Laboratory parameters included fasting plasma glucose, glycated hemoglobin, total cholesterol, triglycerides, high-density lipoprotein cholesterol, low-density lipoprotein cholesterol, and complete blood cell counts. Neutrophil, lymphocyte, and platelet counts were obtained from the first routine blood sample collected at hospital admission. Complete blood cell counts were expressed in ×10^9^/L.

The neutrophil-to-lymphocyte ratio (NLR) was calculated as:


$$\text{NLR}=\frac{\text{neutrophil count}}{\text{lymphocyte count}}$$

The systemic immune-inflammation index was calculated as:


$$\text{SII}=\frac{\text{platelet count}\times \text{neutrophil count}}{\text{lymphocyte count}}$$

For regression analyses, SII was modeled per 100-unit increase, platelet count per 10×10^9^/L increase, and NLR per 1-unit increase.

### 2.5 Exploratory targeted gene-expression analysis

Human sural nerve gene-expression data from the GSE95849 dataset were obtained from the GEO database. The dataset was generated on the GPL22448 platform. The processed expression matrix available in GEO was used for the present analysis together with the corresponding platform annotation information. GSE95849 contains samples from patients with DPN, patients with diabetes without DPN, and healthy controls. For the present targeted analysis, the primary comparison was restricted to six DPN samples (GSM2527034–GSM2527039) and six samples from patients with diabetes without DPN (GSM2527022–GSM2527027), in order to examine gene-expression differences associated with neuropathy within a diabetic background. Healthy-control samples were not included in the primary comparison.

Rather than performing a transcriptome-wide differential-expression analysis, we conducted an exploratory targeted analysis of three prespecified candidate genes, *JUN*, *BDNF*, and *PDGFRA*. Probe identifiers corresponding to the three candidate genes were identified using the GPL22448 platform annotation. When multiple probes corresponded to the same candidate gene, their expression values were averaged to obtain a single gene-level expression value for each sample.

The GEO expression matrix was used as the input for downstream analysis, and no additional global normalization or batch-correction procedure was applied in our analysis pipeline. The expression scale was inspected before statistical analysis; when the maximum expression value exceeded 50, expression values were transformed using log2(x+1). No additional samples were excluded on the basis of outlier removal or model-based quality-control criteria in this targeted analysis.

Expression levels of *JUN*, *BDNF*, and *PDGFRA* were compared between the DPN and diabetes-without-DPN groups using independent two-sample Student’s t-tests. To account for multiple comparisons across the three candidate-gene tests, nominal P values were adjusted using the Benjamini–Hochberg (BH) procedure to control the false-discovery rate. Both nominal and BH-adjusted P values were reported, with an adjusted *P* < 0.05 considered statistically significant for the candidate-gene comparisons. In addition, an exploratory Pearson correlation analysis was performed to assess the relationship between *JUN* and *BDNF* expression among the six DPN samples.

### 2.6 Statistical analysis of the NHANES cohort

Baseline characteristics of the NHANES analytic cohort were summarized using unweighted descriptive statistics. Continuous variables were presented as means ± standard deviations, whereas categorical variables were presented as counts and percentages. Between-group comparisons were performed using Student’s t-test for continuous variables and the chi-square test for categorical variables. Logistic regression was used to evaluate the association between SII and prevalent DPN. SII was analyzed both continuously, per 100-unit increase, and categorically according to quartiles.

Three nested models were constructed. Model 1 was unadjusted. Model 2 was adjusted for age and sex. Model 3 was additionally adjusted for body mass index (BMI) and glycated hemoglobin (HbA1c). In the quartile analysis, the lowest SII quartile was used as the reference group. The P value for trend was calculated by assigning each participant the median SII value of the corresponding quartile and modeling this value as a continuous variable.

### 2.7 Statistical analysis of the external clinical cohort

Continuous variables were summarized as mean ± standard deviation or median (interquartile range), as appropriate according to their distribution, and categorical variables were expressed as number (percentage). Between-group comparisons were performed using Student’s t-test or the Mann–Whitney U test for continuous variables, as appropriate, and the chi-square test or Fisher’s exact test for categorical variables.

SII was calculated as platelet count × neutrophil count/lymphocyte count. Associations between SII and prevalent DPN were evaluated using logistic regression. To facilitate interpretation and numerical stability, SII was modeled per 100-unit increase. Four sequential models were fitted: Model 1 was unadjusted; Model 2 adjusted for age and sex; Model 3 additionally adjusted for body mass index, diabetes duration, and HbA1c; and Model 4 further adjusted for hypertension, diabetic nephropathy, smoking, alcohol consumption, total cholesterol, triglycerides, high-density lipoprotein cholesterol, and low-density lipoprotein cholesterol. Effect estimates are reported as odds ratios (ORs) with 95% confidence intervals (CIs).

Multicollinearity among predictors included in Model 4 was assessed using variance inflation factors (VIFs), with VIF values <5 considered indicative of no substantial multicollinearity.

The functional forms of the associations of SII and platelet count with prevalent DPN were further evaluated using restricted cubic spline analyses with three knots placed at the 10th, 50th, and 90th percentiles of their respective distributions. The median value of each exposure was used as the reference. The spline models were adjusted for the same covariates as Model 4. Overall associations were evaluated using joint Wald tests for the spline terms, whereas nonlinearity was evaluated using likelihood-ratio tests comparing the spline models with the corresponding linear models.

The variables included in the primary multivariable analysis were complete for all 400 participants; therefore, no imputation was performed. An approximate *post hoc* power analysis was additionally conducted using the adjusted log-odds ratio and its standard error derived from the fully adjusted model with a two-sided α level of 0.05. Because observed *post hoc* power is dependent on the observed effect size, ORs and their 95% CIs were considered the primary measures of effect magnitude and precision. All statistical tests were two-sided, and P<0.05 was considered statistically significant.

### 2.8 Statistical software and general considerations

Statistical analyses were performed using Python version 3.13 with pandas, NumPy, SciPy, statsmodels, patsy, scikit-learn, and matplotlib. Conventional logistic regression models were fitted for the NHANES cohort, whereas conventional and bias-reduced logistic regression models were fitted separately for the external clinical cohort.

Firth bias-reduced logistic regression was implemented using a custom penalized-likelihood algorithm with Jeffreys-prior adjustment. Wald-type 95% confidence intervals and two-sided P values were derived from the penalized model. A two-sided *P* value <0.05 was considered statistically significant.

## 3 Results

### 3.1 Population characteristics in NHANES

A total of 555 participants from NHANES 1999–2000 were included in the discovery cohort. Baseline characteristics stratified by DPN status are presented in **Table 1**. Age (*P* = 0.070), sex (*P* = 0.420), race/ethnicity (P = 0.713), BMI (*P* = 0.200), and smoking status (*P* = 0.655) did not differ significantly between participants with and without DPN. Similarly, fasting glucose (*P* = 0.335), HbA1c (*P* = 0.423), total cholesterol (*P* = 0.450), and triglycerides (*P* = 0.596) did not differ significantly between the two groups.

**Table 1:** Baseline characteristics of the NHANES discovery cohort stratified by DPN status.

Variable	Total (N = 555)	Non-DPN (n = 491)	DPN (n = 64)	*P*-value
**Age** (years)	62.7 ± 12.0	62.4 ± 12.0	65.4 ± 12.1	0.070
**Male**, n (%)	308 (55.5%)	276 (56.2%)	32 (50.0%)	0.420
**Race/Ethnicity**, n (%)				0.713
Non-Hispanic White	257 (46.3%)	231 (47.0%)	26 (40.6%)	
Non-Hispanic Black	84 (15.1%)	74 (15.1%)	10 (15.6%)	
Mexican American	170 (30.6%)	149 (30.3%)	21 (32.8%)	
Other Hispanic	33 (5.9%)	27 (5.5%)	6 (9.4%)	
Other/Multi-racial	11 (2.0%)	10 (2.0%)	1 (1.6%)	
**BMI** (kg/m²)	29.7 ± 6.1	29.8 ± 6.2	28.9 ± 5.1	0.200
**Smoking Status**, n (%)				0.655
Never/Former smoker	250 (45.0%)	219 (44.6%)	31 (48.4%)	
Current smoker	305 (55.0%)	272 (55.4%)	33 (51.6%)	
**Blood Pressure** (mmHg)				
Systolic BP	136.9 ± 22.1	136.7 ± 22.1	138.0 ± 22.6	0.672
Diastolic BP	72.1 ± 16.2	72.6 ± 16.0	68.7 ± 17.7	0.126
**Metabolic Profile**				
Fasting Glucose (mg/dL)	128.5 ± 45.3	127.7 ± 44.3	134.3 ± 52.3	0.335
HbA1c (%)	6.2 ± 1.5	6.2 ± 1.4	6.4 ± 1.8	0.423
Total Cholesterol (mg/dL)	212.3 ± 41.0	211.8 ± 40.8	216.1 ± 42.9	0.450
Triglycerides (mg/dL)	184.5 ± 131.5	183.1 ± 124.2	195.4 ± 179.5	0.596
Creatinine (mg/dL)	0.8 ± 0.5	0.8 ± 0.5	0.8 ± 0.3	0.791
**Inflammatory Markers**				
Platelet Count (×10^9^/L)	258.1 ± 66.6	260.3 ± 67.2	241.0 ± 59.2	**0.018**
SII (×10^9^/L)	593.7 ± 371.3	605.2 ± 381.7	505.5 ± 265.0	**0.009**

Data are presented as unweighted means ± standard deviations (SDs) for continuous variables and unweighted counts (percentages) for categorical variables. BMI, body mass index; DPN, diabetic peripheral neuropathy; HbA1c, glycated hemoglobin; SII, systemic immune-inflammation index. P-values were calculated using Student’s t-test for continuous variables and the Chi-square test for categorical variables. Bold values indicate statistical significance (P < 0.05).

In contrast, participants with DPN had lower platelet counts than those without DPN (241.0 ± 59.2 vs. 260.3 ± 67.2 × 10^9^/L, P = 0.018). SII was also lower in participants with DPN (505.5 ± 265.0 vs. 605.2 ± 381.7 × 10^9^/L, P = 0.009).

### 3.2 Inverse association between SII and prevalent DPN in NHANES

In the NHANES cohort, multivariable logistic regression analyses showed an inverse association between SII and prevalent DPN (**Supplementary Table 1**). In the fully adjusted model (Model 3), which adjusted for age, sex, BMI, and HbA1c, each 100-unit increase in SII was associated with lower odds of prevalent DPN (OR = 0.886, 95% CI: 0.798–0.983; P = 0.023).

**Figure 2:**
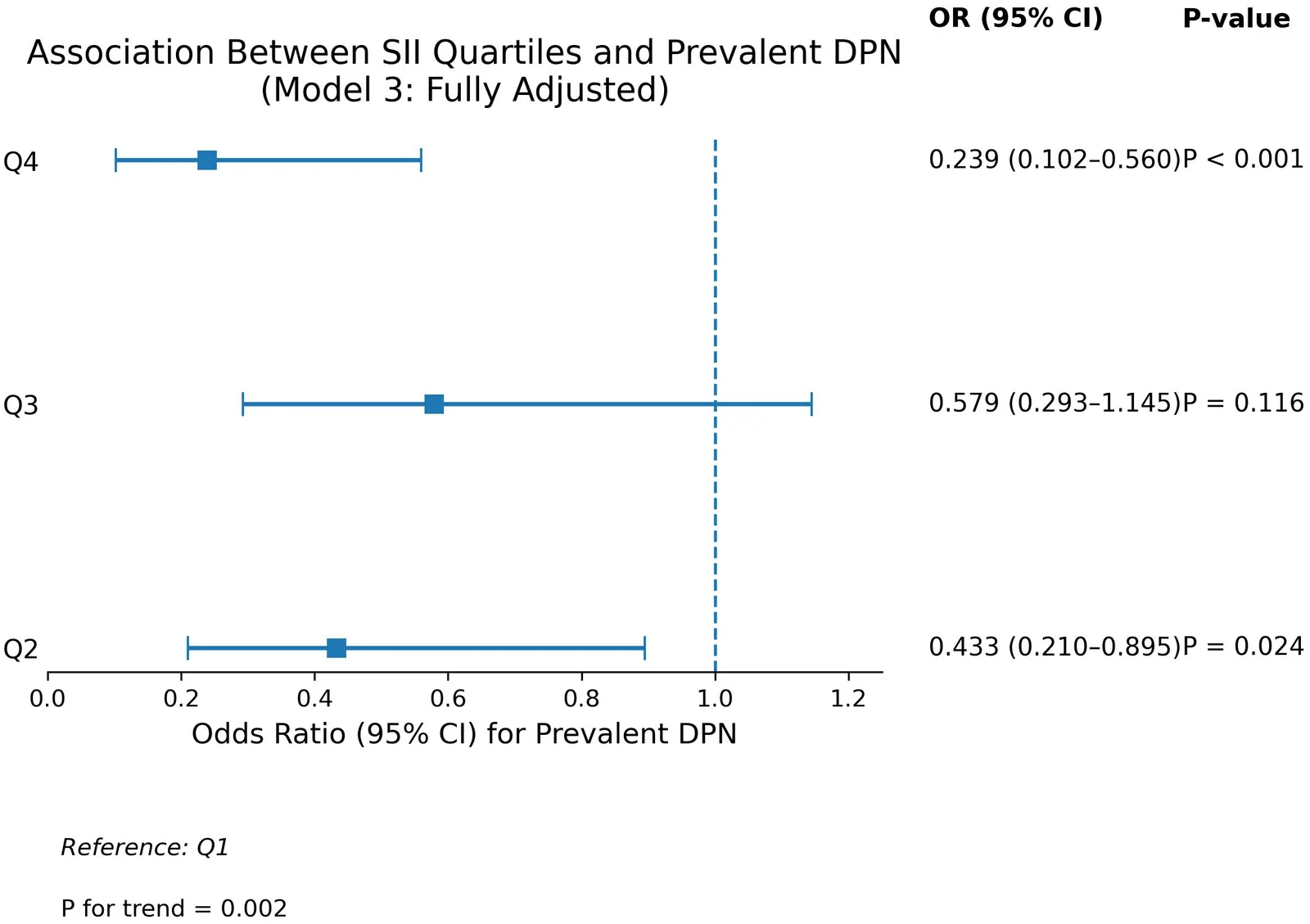
Association between SII quartiles and prevalent DPN in the NHANES discovery cohort. Odds ratios (ORs) and 95% confidence intervals (CIs) for prevalent DPN are shown across SII quartiles, with the lowest quartile (Q1) as the reference. Estimates were derived from the fully adjusted Model 3. Compared with Q1, participants in Q4 had lower odds of prevalent DPN (OR = 0.239, 95% CI: 0.102–0.560; *P* < 0.001). The P value for trend across SII quartiles was 0.002. SII, systemic immune-inflammation index; DPN, diabetic peripheral neuropathy; OR, odds ratio; CI, confidence interval.

When SII was analyzed in quartiles, participants in the highest quartile (Q4) had lower odds of prevalent DPN than those in the lowest quartile (Q1) (OR = 0.239, 95% CI: 0.102–0.560; *P* < 0.001; **Figure 2**). An inverse trend across increasing SII quartiles was also observed (*P* for trend = 0.002), supporting a graded inverse association between SII and prevalent DPN.

### 3.3 Exploratory targeted gene-expression analysis of sural nerve tissue (GEO dataset)

To provide exploratory biological context, we performed a targeted gene-expression analysis using human sural nerve data from the GSE95849 dataset. The primary comparison included six DPN samples and six samples from patients with diabetes without DPN and focused on three candidate genes, *JUN*, *BDNF*, and *PDGFRA*, selected for their potential relevance to nerve-injury responses, neurotrophic signaling, and platelet-related biological processes.

**Figure 3:**
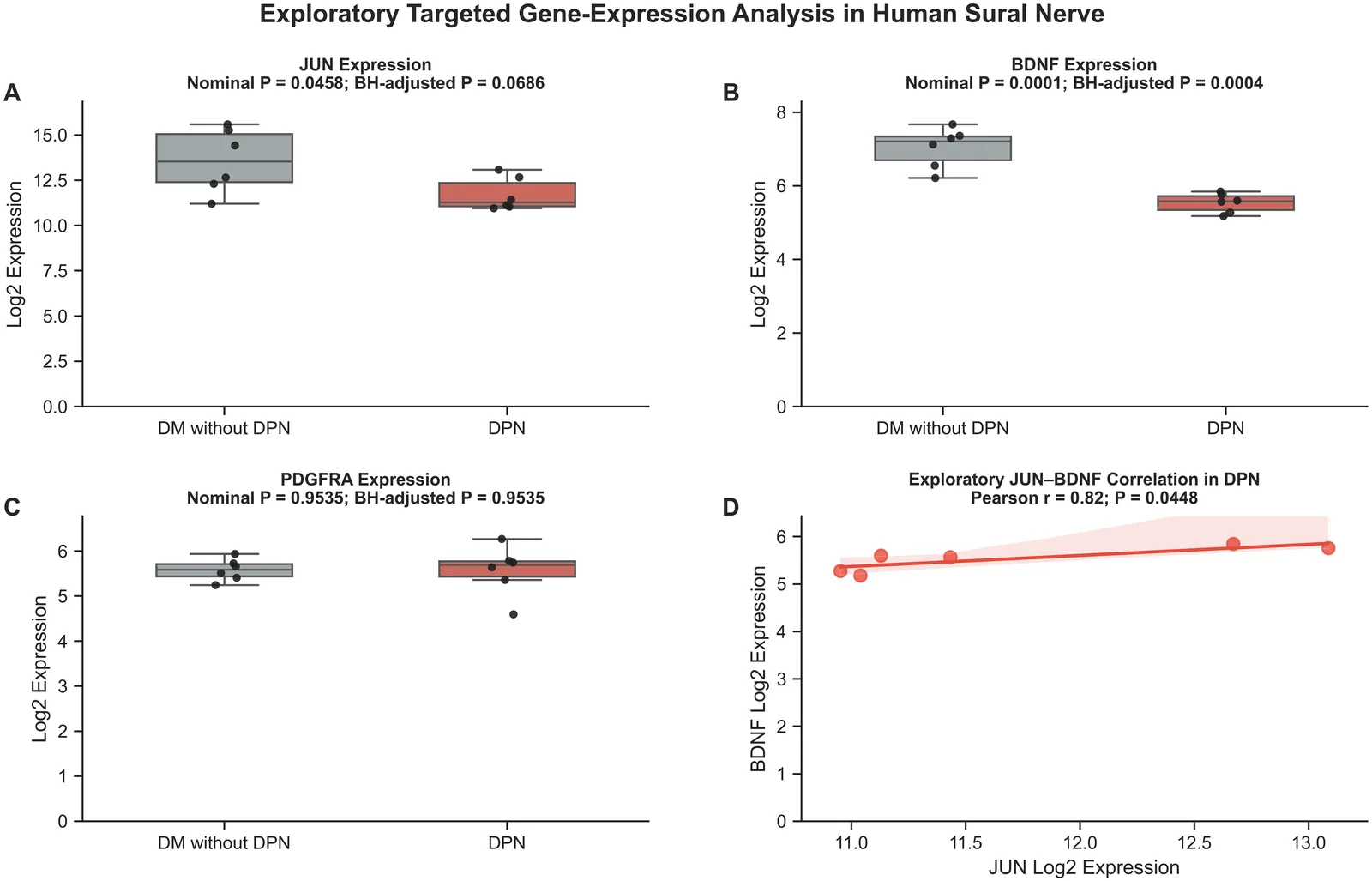
Exploratory targeted gene-expression analysis in human sural nerve samples from the GSE95849 dataset. **(A–C)** Expression levels of JUN, BDNF, and PDGFRA were compared between six DPN samples and six samples from patients with diabetes without DPN. BDNF expression was lower in DPN samples (5.538 ± 0.262 vs. 7.037 ± 0.546; nominal P = 0.000121; BH-adjusted P = 0.000364). JUN expression was also lower in DPN samples at the nominal level (11.718 ± 0.922 vs. 13.578 ± 1.773; nominal P = 0.045761), but the difference did not remain statistically significant after BH correction (BH-adjusted P = 0.068641). PDGFRA expression did not differ between groups (5.566 ± 0.558 vs. 5.581 ± 0.245; nominal and BH-adjusted P = 0.953521). Individual points represent individual sural nerve samples, and box plots summarize the expression distributions. Between-group comparisons were performed using independent two-sample Student’s t-tests. **(D)** Exploratory Pearson correlation analysis among the six DPN samples showed a positive correlation between JUN and BDNF expression (r = 0.82, P = 0.0448). Given the small sample size, the correlation should be interpreted cautiously and does not establish coordinated molecular regulation. These findings are exploratory and hypothesis-generating. BH, Benjamini–Hochberg; DPN, diabetic peripheral neuropathy.

*BDNF* expression was significantly lower in DPN samples than in diabetes-without-DPN samples (5.538 ± 0.262 vs. 7.037 ± 0.546; nominal *P* = 0.000121; BH-adjusted *P* = 0.000364; **Figure 3B**). *JUN* expression was also lower in DPN samples (11.718 ± 0.922 vs. 13.578 ± 1.773; nominal *P* = 0.045761); however, this difference did not remain statistically significant after Benjamini–Hochberg correction (BH-adjusted *P* = 0.068641; **Figure 3A**). *PDGFRA* expression did not differ between the two groups (5.566 ± 0.558 vs. 5.581 ± 0.245; nominal and BH-adjusted *P* = 0.953521; **Figure 3C**).

Among the six DPN samples, exploratory Pearson correlation analysis showed a positive correlation between *JUN* and *BDNF* expression (r = 0.82, *P* = 0.0448; **Figure 3D**). Given the very small sample size, this correlation should be interpreted cautiously and does not establish coordinated molecular regulation or a specific mechanistic pathway. Overall, these targeted findings provide preliminary biological context for differences in neurotrophic and injury-responsive gene expression in DPN but should be regarded as exploratory and hypothesis-generating. Detailed expression estimates and nominal and BH-adjusted P values for the three candidate genes are provided in **Supplementary Table 6**.

### 3.4 External validation in an independent clinical cohort

The external clinical cohort comprised 400 patients with T2DM, including 110 patients without DPN and 290 patients with DPN, from the Department of Neurosurgery, China-Japan Friendship Hospital. Patients with and without DPN did not differ significantly in age (62.0 ± 9.2 vs. 61.1 ± 10.4 years, *P* = 0.379) or diabetes duration (14.9 ± 8.3 vs. 13.9 ± 8.1 years, *P* = 0.265) (**Supplementary Table 2**).

**Figure 4:**
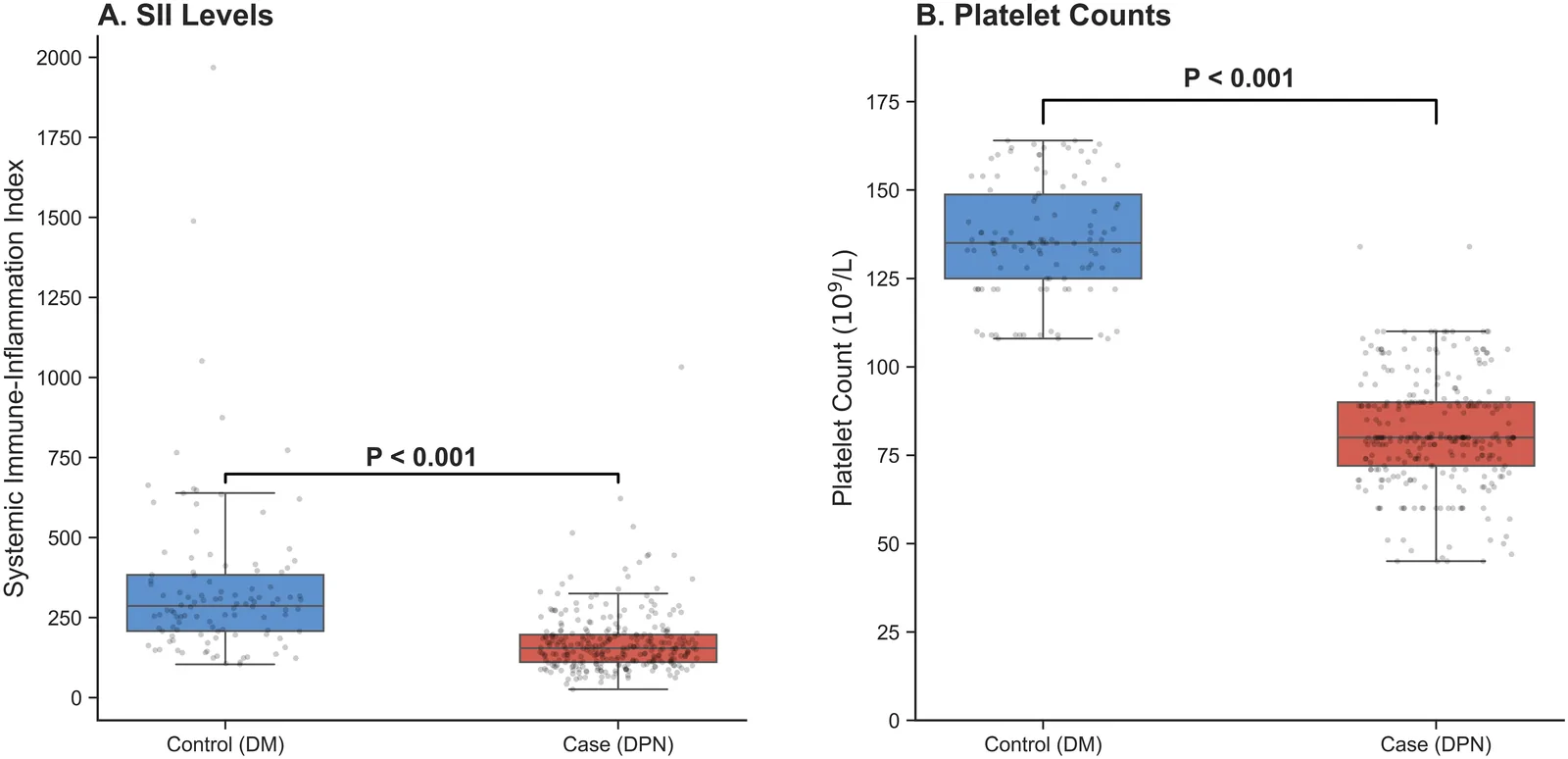
Hematological comparisons in the external clinical cohort. SII **(A)** and platelet count **(B)** were compared between patients with T2DM without DPN (n = 110) and patients with DPN (n = 290) at China-Japan Friendship Hospital. Platelet counts are expressed in ×10^9^/L. Data are presented as box plots with individual data points overlaid. Between-group comparisons were performed using independent two-sample Student’s t-tests; both SII and platelet count were lower in the DPN group (*P* < 0.001 for both comparisons). DPN, diabetic peripheral neuropathy; SII, systemic immune-inflammation index; T2DM, type 2 diabetes mellitus.

Consistent with the direction of the association observed in NHANES, patients with DPN had substantially lower platelet counts than those without DPN (81.6 ± 15.5 vs. 135.9 ± 16.3 × 10^9^/L, P < 0.001; **Figure 4B**). SII was also significantly lower in patients with DPN (172.1 ± 99.4 vs. 344.3 ± 257.7 × 10^9^/L, P < 0.001; **Figure 4A**).

### 3.5 Adjusted association, nonlinearity, and component analyses

Multivariable logistic regression analyses in the external clinical cohort consistently showed an inverse association between SII and prevalent DPN across sequentially adjusted models (**Supplementary Table 3**). In the fully adjusted Model 4, which accounted for age, sex, BMI, diabetes duration, HbA1c, hypertension, diabetic nephropathy, smoking, alcohol consumption, total cholesterol, triglycerides, high-density lipoprotein cholesterol, and low-density lipoprotein cholesterol, each 100-unit increase in SII corresponded to an OR of 0.396 (95% CI: 0.304–0.516; *P* < 0.001) for prevalent DPN.

No substantial multicollinearity was identified among predictors included in Model 4. Variance inflation factor values ranged from 1.028 to 1.238, with the highest value observed for smoking (VIF = 1.238).

Restricted cubic spline analysis further showed a significant overall association between SII and prevalent DPN (*P* for overall association < 0.001), together with evidence of nonlinearity (*P* for nonlinearity = 0.00047 by likelihood-ratio test). Using the median SII (174.7) as the reference, the inverse association was more pronounced at lower SII levels and gradually attenuated at higher SII levels (**Supplementary Figure 1**).

We next examined the individual hematological components underlying SII in fully adjusted models (**Supplementary Table 4**). When modeled separately, platelet count showed a large inverse association with prevalent DPN (OR = 0.065 per 10 × 10^9^/L increase, 95% CI: 0.024–0.175; P < 0.001). NLR showed a more modest inverse association (OR = 0.799 per 1-unit increase, 95% CI: 0.665–0.960; P = 0.016). Neutrophil count was not significantly associated with prevalent DPN (OR = 1.046 per 1 × 10^9^/L increase, 95% CI: 0.894–1.225; P = 0.573), whereas lymphocyte count showed a weak positive association (OR = 1.461 per 1 × 10^9^/L increase, 95% CI: 1.006–2.124; P = 0.047).

In the mutually adjusted model including platelet count and NLR, the platelet-count association remained large (OR = 0.055 per 10 × 10^9^/L increase, 95% CI: 0.018–0.167; P < 0.001), whereas the association with NLR was attenuated and was no longer statistically significant (OR = 0.695 per 1-unit increase, 95% CI: 0.441–1.095; P = 0.117). Thus, among the hematological components examined, platelet count showed the strongest and most consistent association with prevalent DPN. These component-level findings are associative and should not be interpreted as evidence that platelet alterations causally mediate the association between SII and DPN.

Given the large magnitude of the platelet-count estimate, additional sensitivity analyses were performed (**Supplementary Table 5**). In Firth bias-reduced logistic regression, platelet count remained inversely associated with prevalent DPN (OR = 0.119 per 10 × 10^9^/L increase, 95% CI: 0.062–0.227; P < 0.001), whereas NLR was not statistically significant (OR = 0.755 per 1-unit increase, 95% CI: 0.515–1.107; P = 0.151). Excluding participants with platelet counts <50 × 10^9^/L did not materially alter the overall findings. After exclusion of participants with platelet counts <100 × 10^9^/L, the association between SII and prevalent DPN was attenuated but remained statistically significant (OR = 0.624 per 100-unit increase, 95% CI: 0.441–0.883; P = 0.008). In the corresponding mutually adjusted component model, platelet count remained inversely associated with prevalent DPN (OR = 0.074 per 10 × 10^9^/L increase, 95% CI: 0.025–0.220), whereas NLR was not statistically significant (OR = 0.656 per 1-unit increase, 95% CI: 0.396–1.087; P = 0.102).

Re-examination of the original clinical laboratory records confirmed that platelet counts were recorded in ×10^9^/L and that the values used in the analyses were consistent with the source records, with no identified unit-conversion or data-entry errors. An additional platelet-specific restricted cubic spline analysis showed a significant overall association between platelet count and prevalent DPN (P < 0.001), with evidence of nonlinearity (P for nonlinearity = 0.002; **Supplementary Figure 2**). The 10th, 50th, and 90th percentiles of platelet count were 66, 89, and 138 ×10^9^/L, respectively, with the median value (89 ×10^9^/L) used as the reference. Given the limited overlap in platelet-count distributions between the DPN and diabetes-without-DPN groups, the magnitude and shape of the spline association were interpreted cautiously.

Finally, based on the effect estimate from the fully adjusted model, an approximate *post hoc* power assessment suggested >99% power for the primary SII association. Because observed *post hoc* power is dependent on the observed effect size, the OR and its 95% CI were considered the primary measures of effect magnitude and precision.

## 4 Discussion

In the present study, lower SII was consistently associated with prevalent DPN in both the NHANES discovery cohort and an independent hospital-based clinical cohort. In the external cohort, this association persisted after multivariable adjustment and showed a significant nonlinear pattern, with a stronger inverse association at lower SII levels and progressive attenuation at higher levels. Component analyses further indicated that platelet count showed the strongest and most consistent association with prevalent DPN among the hematological components examined. In parallel, exploratory targeted gene-expression analysis of human sural nerve tissue showed significantly lower *BDNF* expression in DPN samples than in diabetes-without-DPN samples. *JUN* expression was also lower at the nominal level, although this difference did not remain significant after BH correction, whereas *PDGFRA* expression did not differ between groups. Taken together, these findings support a reproducible association between lower SII and prevalent DPN and provide exploratory biological context, but they do not establish a causal or mechanistic link between circulating platelet-related indices and nerve-tissue molecular alterations.

The inverse association observed in our study differs from previous reports linking higher SII to DPN (6). Several factors may contribute to these apparently divergent findings. First, differences in population characteristics, including ethnicity, age distribution, diabetes duration, disease severity, and comorbidity burden, may modify the relationship between systemic hematological indices and neuropathy. Second, differences in DPN ascertainment and the distribution of disease stages across studies may contribute to heterogeneity, although the present cross-sectional data cannot establish stage-dependent temporal changes in SII. Third, medication use, including glucose-lowering, lipid-lowering, antiplatelet, and other therapies, may influence circulating blood-cell counts or platelet activity and may differ substantially across study populations. Fourth, platelet count does not necessarily reflect platelet functional status; changes in platelet activation, turnover, or function could potentially contribute to discrepancies between studies even when platelet counts are similar. Finally, pre-analytical conditions, laboratory platforms, blood-count measurement procedures, and between-study variability in SII distributions may also influence effect estimates. In addition, our restricted cubic spline analysis demonstrated significant nonlinearity in the external cohort, suggesting that a single linear effect estimate may not fully characterize the SII–DPN relationship across the entire exposure range. These possibilities remain speculative and require direct evaluation in harmonized prospective cohorts.

The prominent platelet association observed in the external cohort deserves particular attention but also requires cautious interpretation. Platelet count showed a substantially stronger association with prevalent DPN than did neutrophil count, lymphocyte count, or NLR. Moreover, the direction of the platelet association remained consistent in Firth bias-reduced logistic regression and in sensitivity analyses excluding participants with very low platelet counts. Nevertheless, the magnitude of the platelet effect estimates was unusually large, and these findings should not be interpreted as demonstrating that reduced platelet count directly causes or mediates DPN. Platelet count may reflect a broader combination of hematological, metabolic, vascular, inflammatory, nutritional, treatment-related, or disease-severity factors that were not fully captured in the present analyses. Independent replication in larger and more diverse populations is therefore needed.

The additional platelet-specific restricted cubic spline analysis also provided evidence of nonlinearity, suggesting that the association may not be adequately summarized by a single linear OR. However, the limited overlap in platelet-count distributions between the DPN and diabetes-without-DPN groups, together with greater uncertainty toward the distributional extremes, further supports cautious interpretation of the magnitude and shape of this association.

Platelets have biological functions extending beyond hemostasis, including participation in inflammatory signaling, endothelial interactions, tissue repair, and the storage and release of several growth factors. *BDNF* is among the mediators that can be stored and released by platelets (15, 16). These properties provide a plausible biological context in which platelet-related processes could be relevant to peripheral nerve injury. However, the present study did not measure circulating *BDNF* concentrations, platelet BDNF content, platelet activation or function, or longitudinal changes in platelet number. Accordingly, our data do not demonstrate that lower platelet counts result in reduced neurotrophic support, nor do they establish a direct platelet–*BDNF* pathway in DPN. The epidemiological findings should therefore be interpreted as hypothesis-generating rather than as evidence of platelet-mediated neurotrophic failure.

The exploratory nerve-tissue findings also require careful interpretation. BDNF expression was lower in DPN nerve samples than in samples from patients with diabetes without DPN, and this difference remained significant after correction for multiple comparisons. This finding is compatible with differences in neurotrophic signaling in established DPN; however, tissue-level BDNF expression does not directly measure circulating BDNF availability, platelet-derived BDNF release, or functional neurotrophic support. Therefore, the present data cannot establish that reduced platelet count leads to lower nerve-tissue BDNF expression or that a platelet–BDNF pathway mediates DPN. JUN has established roles in Schwann-cell reprogramming after peripheral nerve injury (17). In the present targeted analysis, JUN expression was lower in DPN samples at the nominal significance level, but the difference did not remain statistically significant after BH correction. Accordingly, the JUN finding should be regarded as suggestive rather than definitive.

More broadly, distal axonal degeneration in diabetic neuropathy has been discussed within “dying-back” models involving impaired axonal maintenance and transport processes (2). Whether the platelet and gene-expression patterns observed in the present study are related to such mechanisms remains unknown. Although *JUN* and *BDNF* expression showed a positive exploratory correlation among the six DPN samples (r = 0.82, *P* = 0.0448), the very small sample size limits the stability and interpretation of this estimate; the correlation should therefore not be considered evidence of a coordinated molecular program or mechanistic interaction. *PDGFRA*, which has been used as a marker associated with mesenchymal and stromal cell populations (18), did not differ significantly between groups. However, the absence of a difference in a single candidate marker cannot exclude broader alterations in stromal composition, fibrosis, or tissue remodeling. Thus, the targeted gene-expression results are best considered preliminary, hypothesis-generating observations that require validation in larger transcriptomic datasets, cell-specific studies, and experimental models.

From a clinical perspective, SII is inexpensive and readily obtainable from routine complete blood counts, which makes the observed association potentially relevant for future clinical research. However, the present study was designed to evaluate association rather than diagnostic or predictive performance. Therefore, SII should not currently be regarded as a validated biomarker, screening tool, or predictor of DPN, and the nonlinear association observed in the external cohort should not be interpreted as defining a clinically actionable threshold or cutoff. Prospective studies are required to determine whether SII provides incremental information beyond established clinical risk factors and whether longitudinal changes in SII are associated with subsequent DPN development or progression. Likewise, the present findings do not provide evidence to support platelet-directed therapy or exogenous neurotrophic supplementation.

## 5 Strengths & limitations

The principal strength of this study is its multidimensional validation strategy, combining the NHANES cohort with detailed clinical information from an independent hospital-based cohort. The exploratory targeted gene-expression analysis of human sural nerve tissue also provides complementary biological context for the observed epidemiological associations.

Several limitations should nevertheless be acknowledged. First, the cross-sectional and observational designs preclude causal inference and do not permit assessment of the temporal relationship between SII and DPN. Longitudinal studies are needed to determine whether changes in SII precede the development or progression of DPN. Moreover, although restricted cubic spline analysis identified a nonlinear association between SII and prevalent DPN in the external cohort, this finding should be interpreted as a description of the exposure–outcome relationship rather than evidence of a specific biological threshold, clinical cutoff, or causal dose–response relationship.

Second, the external cohort was retrospectively assembled from a single hospital, which may limit the generalizability of the findings and introduce selection bias. Formal blinding procedures were not implemented, and DPN status was ascertained from pre-existing clinical diagnoses documented in electronic medical records rather than through standardized prospective adjudication. Although SII was calculated retrospectively and was not used to determine DPN status, treating clinicians may have had access to routine laboratory results during clinical care; therefore, potential observer bias cannot be completely excluded. Moreover, nerve conduction studies and validated diagnostic scoring instruments were not systematically applied to all participants, and heterogeneity in routine clinical assessment may have introduced some degree of diagnostic misclassification. In the NHANES cohort, DPN was operationally defined using monofilament-detected loss of protective sensation, which may not capture the full clinical spectrum of DPN, particularly early or small-fiber neuropathy.

Third, despite multivariable adjustment, residual confounding cannot be excluded. Information regarding specific glucose-lowering medications, insulin therapy, statin use, antiplatelet or other platelet-modifying medications, nutritional status or deficiencies, detailed quantitative renal-function measures, and other factors that may influence blood-cell counts or neuropathy status was unavailable or incomplete for consistent adjustment.

Fourth, the gene-expression analysis was exploratory and restricted to selected candidate genes rather than constituting a comprehensive transcriptome-wide or mechanistic investigation. Although platelets are known to store and release *BDNF* (16, 19, 20), circulating *BDNF* concentrations, platelet *BDNF* content, platelet function, and the specific cellular sources of nerve-tissue *BDNF* were not directly assessed. Lower *BDNF* expression in DPN nerve tissue is compatible with differences in neurotrophic signaling; however, tissue-level *BDNF* expression does not directly represent circulating *BDNF* availability, platelet *BDNF* content, or functional neurotrophic support. Moreover, the targeted analysis included only six DPN and six diabetes-without-DPN samples, substantially limiting statistical precision and generalizability. Therefore, platelet count should not be regarded as a validated surrogate for systemic or neural neurotrophic capacity on the basis of the present data, and the biological interpretation requires validation in larger molecular datasets and experimental studies.

Finally, no *a priori* sample-size calculation was performed because the external cohort retrospectively included all eligible patients identified during the predefined study period. Although an approximate *post hoc* analysis suggested high statistical power for the primary association between SII and DPN, *post hoc* power is intrinsically dependent on the observed effect size and does not replace prospective sample-size planning. The present study may also have limited statistical power for secondary subgroup or interaction analyses. Larger multicenter prospective studies incorporating standardized neuropathy phenotyping, more complete medication and renal-function data, and direct measurements of platelet function and circulating neurotrophic factors are warranted.

## 6 Conclusions

Lower SII was consistently associated with prevalent DPN across the NHANES discovery cohort and an independent external clinical cohort. In the external cohort, the association was nonlinear, and platelet count showed the strongest and most consistent association among the hematological components examined. Exploratory targeted gene-expression findings in sural nerve tissue provide complementary biological context indicating differences in neurotrophic and injury-responsive gene expression between DPN and diabetes without DPN; however, these observations do not establish a causal or mechanistic relationship between platelet-related hematological indices and DPN. Prospective multicenter studies and larger molecular datasets are needed to clarify the temporal relationship, potential clinical utility, and underlying biological processes.

## Data Availability

The original contributions presented in the study are included in the article/**Supplementary Material**. Further inquiries can be directed to the corresponding authors.
