# Supplementary material for: Systemic immune-inflammation index is associated with diabetic peripheral neuropathy: evidence from NHANES, an external clinical cohort, and exploratory human nerve gene-expression analysis
Source: Front Endocrinol (Lausanne). 2026 Sep 7;17:1948081. doi: 10.3389/fendo.2026.1948081 (PMC13590368; doi:10.3389/fendo.2026.1948081)
Supplement: Supplementary file 1 [file SupplementaryFile1.docx]

Supplementary Table 1. Logistic regression analyses of the association between SII and prevalent diabetic peripheral neuropathy in the NHANES 1999–2000 discovery cohort

| SII analysis | SII range | DPN events, n/N (%) | Odds Ratio (95% CI) | P-value |
| --- | --- | --- | --- | --- |
| Continuous SII (per 100-unit increase) |  |  |  |  |
| Model 1 | — | 64/555 (11.5%) | 0.896 (0.810–0.992) | 0.034 |
| Model 2 | — | 64/555 (11.5%) | 0.882 (0.794–0.980) | 0.019 |
| Model 3 | — | 63/551 (11.4%) | 0.886 (0.798–0.983) | 0.023 |
| Categorical SII (quartiles) |  |  |  |  |
| Q1 (Lowest) | 56.4–369.1 | 25/137 (18.2%) | 1.00 (Reference) | — |
| Q2 | 369.5–510.0 | 13/138 (9.4%) | 0.433 (0.210–0.895) | 0.024 |
| Q3 | 510.9–738.2 | 17/138 (12.3%) | 0.579 (0.293–1.145) | 0.116 |
| Q4 (Highest) | 739.1–4930.0 | 8/138 (5.8%) | 0.239 (0.102–0.560) | **<0.001** |
| P for trend | — | — | — | 0.002 |

**Note:** Analyses were performed using conventional unweighted logistic regression. SII was modeled continuously per 100-unit increase and categorically according to quartiles, with Q1 as the reference category. The quartile cut points in the full discovery cohort were 369.316, 510.000, and 738.651. Model 1 was unadjusted; Model 2 was adjusted for age and sex; and Model 3 was additionally adjusted for body mass index and HbA1c. Four participants with missing body mass index were excluded from Model 3, resulting in an analytic sample of 551 participants (63 DPN cases). Quartile-specific event counts correspond to the Model 3 analytic sample. The P value for trend was calculated by assigning the median SII value of each quartile to participants in that quartile and modeling this variable continuously in Model 3. CI, confidence interval; DPN, diabetic peripheral neuropathy; HbA1c, glycated hemoglobin; OR, odds ratio; SII, systemic immune-inflammation index.

Supplementary Table 2. Baseline characteristics of the external clinical validation cohort (China-Japan Friendship Hospital)

| **Variable** | **Control (DM)(n = 110)** | **Case (DPN)(n = 290)** | ***P*-value** |
| --- | --- | --- | --- |
| **Age** (years) | 62.0 ± 9.2 | 61.1 ± 10.4 | 0.379 |
| **Male**, n (%) | 78 (70.9%) | 193 (66.6%) | 0.476 |
| **BMI** (kg/m²) | 24.4 ± 3.3 | 24.5 ± 3.9 | 0.796 |
| **Diabetes Duration** (years) | 14.9 ± 8.3 | 13.9 ± 8.1 | 0.265 |
| **Metabolic Profile** |  |  |  |
| **HbA1c** (%) | 7.0 ± 1.6 | 7.4 ± 2.0 | 0.042 |
| **Inflammatory Markers** |  |  |  |
| **Platelet Count** (10⁹/L) | 135.9 ± 16.3 | 81.6 ± 15.5 | **< 0.001** |
| **SII** (10⁹/L) | 344.3 ± 257.7 | 172.1 ± 99.4 | **< 0.001** |

**Note:**Data are presented as mean ± standard deviation (SD) for continuous variables and number (percentage) for categorical variables. Differences between groups were analyzed using Student’s *t*-test (or Mann-Whitney U test) for continuous variables and Chi-square test for categorical variables. **Abbreviations:** *BMI*, Body Mass Index; *DM*, Diabetes Mellitus; *DPN*, Diabetic Peripheral Neuropathy; *HbA1c*, Glycated Hemoglobin; *SII*, Systemic Immune-Inflammation Index. Bold *P*-values indicate statistical significance (***P* < 0.05**).

Supplementary Table 3. Multivariable logistic regression analysis of the association between SII and prevalent DPN in the clinical validation cohort

| **Model** | **OR (95% CI)^a^** | ***P*-value** |
| --- | --- | --- |
| **Model 1** (Unadjusted) | 0.390 (0.304–0.499) | **< 0.001** |
| **Model 2** (Adjusted for age and sex) | 0.389 (0.303–0.500) | **< 0.001** |
| **Model 3** (Further adjusted) | 0.376 (0.291–0.485) | **< 0.001** |
| **Model 4** (Fully adjusted) | 0.396 (0.304–0.516) | **< 0.001** |

**Note:**Odds ratios represent the association with prevalent DPN per 100-unit increase in SII. Model 1 was unadjusted. Model 2 was adjusted for age and sex. Model 3 was additionally adjusted for body mass index, diabetes duration, and HbA1c. Model 4 was further adjusted for hypertension, diabetic nephropathy, smoking, alcohol consumption, total cholesterol, triglycerides, high-density lipoprotein cholesterol, and low-density lipoprotein cholesterol. CI, confidence interval; DPN, diabetic peripheral neuropathy; SII, systemic immune-inflammation index.

Supplementary Table 4. Associations of SII and its hematological components with prevalent diabetic peripheral neuropathy in the clinical validation cohort

| **Part A. Individual marker models** | | |
| --- | --- | --- |
| **Marker** | **OR (95% CI)** | **P-value** |
| SII, per 100-unit increase | 0.396 (0.304–0.516) | **<0.001** |
| Platelet count, per 10 ×10⁹/L increase | 0.065 (0.024–0.175) | **<0.001** |
| NLR, per 1-unit increase | 0.799 (0.665–0.960) | 0.016 |
| Neutrophil count, per 1 ×10⁹/L increase | 1.046 (0.894–1.225) | 0.573 |
| Lymphocyte count, per 1 ×10⁹/L increase | 1.461 (1.006–2.124) | 0.047 |
| **Part B. Mutually adjusted platelet–NLR model** | | |
| **Marker** | **OR (95% CI)** | **P-value** |
| Platelet count, per 10 ×10⁹/L increase | 0.055 (0.018–0.167) | **<0.001** |
| NLR, per 1-unit increase | 0.695 (0.441–1.095) | 0.117 |

**Note:** In Part A, each hematological marker was entered separately into a fully adjusted logistic regression model. In Part B, platelet count and NLR were entered simultaneously into the same model. All models were adjusted for age, sex, body mass index, diabetes duration, HbA1c, hypertension, diabetic nephropathy, smoking, alcohol consumption, total cholesterol, triglycerides, high-density lipoprotein cholesterol, and low-density lipoprotein cholesterol. ORs represent the odds of prevalent DPN per specified unit increase in each marker. CI, confidence interval; DPN, diabetic peripheral neuropathy; NLR, neutrophil-to-lymphocyte ratio; SII, systemic immune-inflammation index.

Supplementary Table 5. Sensitivity analyses of the associations of SII, platelet count, and NLR with prevalent diabetic peripheral neuropathy in the clinical validation cohort

| **Analysis** | **Marker** | **OR (95% CI)** | **P-value** |
| --- | --- | --- | --- |
| Firth logistic regression | Platelet count | 0.119 (0.062–0.227) | **<0.001** |
| Firth logistic regression | NLR | 0.755 (0.515–1.107) | 0.151 |
| Excluding PLT <50 | SII | 0.400 (0.307–0.521) | **<0.001** |
| Excluding PLT <100 | SII | 0.624 (0.441–0.883) | 0.008 |
| Excluding PLT <100, joint model | Platelet count | 0.074 (0.025–0.220) | **<0.001** |
| Excluding PLT <100, joint model | NLR | 0.656 (0.396–1.087) | 0.102 |

**Note:** All standard logistic regression models were adjusted for age, sex, body mass index, diabetes duration, HbA1c, hypertension, diabetic nephropathy, smoking, alcohol consumption, total cholesterol, triglycerides, high-density lipoprotein cholesterol, and low-density lipoprotein cholesterol. Firth bias-reduced logistic regression was used to account for potential small-sample and separation bias in the mutually adjusted platelet–NLR model. ORs represent the odds of prevalent DPN per 100-unit increase in SII, per 10×10⁹/L increase in platelet count, or per 1-unit increase in NLR. CI, confidence interval; DPN, diabetic peripheral neuropathy; NLR, neutrophil-to-lymphocyte ratio; SII, systemic immune-inflammation index.

Supplementary Table 6. Results of the exploratory targeted gene-expression analysis after Benjamini–Hochberg correction

| **Gene** | **DPN, mean ± SD** | **DM without DPN, mean ± SD** | **Nominal P value** | **BH-adjusted P value** | **Statistical interpretation** |
| --- | --- | --- | --- | --- | --- |
| JUN | 11.718 ± 0.922 | 13.578 ± 1.773 | 0.045761 | 0.068641 | Not significant after BH correction |
| BDNF | 5.538 ± 0.262 | 7.037 ± 0.546 | 0.000121 | 0.000364 | Significant after BH correction |
| PDGFRA | 5.566 ± 0.558 | 5.581 ± 0.245 | 0.953521 | 0.953521 | Not significant |

**Note:** Expression levels were compared between six diabetic peripheral neuropathy (DPN) samples and six samples from patients with diabetes without DPN using independent two-sample Student’s t-tests. Nominal P values from the three prespecified candidate-gene comparisons were adjusted using the Benjamini–Hochberg (BH) procedure to control the false-discovery rate. A BH-adjusted P value <0.05 was considered statistically significant. Although JUN showed a nominal between-group difference, this finding did not remain statistically significant after BH correction. BDNF remained significant after multiple-comparison correction, whereas PDGFRA showed no significant between-group difference.

**Supplementary Figure S1.** Restricted cubic spline analysis of the association between SII and prevalent DPN in the external clinical cohort. Restricted cubic spline regression with three knots placed at the 10th, 50th, and 90th percentiles of the SII distribution was fitted within the fully adjusted logistic regression model. The solid line represents the estimated odds ratio for prevalent DPN, and the shaded area represents the 95% confidence interval. The median SII (174.735) was used as the reference (OR = 1.0). The model was adjusted for age, sex, body mass index, diabetes duration, HbA1c, hypertension, diabetic nephropathy, smoking, alcohol consumption, total cholesterol, triglycerides, high-density lipoprotein cholesterol, and low-density lipoprotein cholesterol. P for overall association was derived from a joint Wald test, and P for nonlinearity was derived from a likelihood-ratio test comparing the spline and linear models.

**Supplementary Figure S2.** Restricted cubic spline analysis of the association between platelet count and prevalent DPN in the external clinical cohort. Restricted cubic spline regression with three knots placed at the 10th, 50th, and 90th percentiles of the platelet-count distribution was fitted within the fully adjusted logistic regression model. The solid line represents the estimated odds ratio for prevalent DPN, and the shaded area represents the 95% confidence interval. The median platelet count (89 ×10⁹/L) was used as the reference (OR = 1.0). The model was adjusted for age, sex, body mass index, diabetes duration, HbA1c, hypertension, diabetic nephropathy, smoking, alcohol consumption, total cholesterol, triglycerides, high-density lipoprotein cholesterol, and low-density lipoprotein cholesterol. P for overall association was derived from a joint Wald test, and P for nonlinearity was derived from a likelihood-ratio test comparing the spline and linear models. The overall association was statistically significant (P < 0.001), with evidence of nonlinearity (P for nonlinearity = 0.002).
